# Mechanisms of acquired resistance to rapalogs in metastatic renal cell carcinoma

**DOI:** 10.1371/journal.pgen.1007679

**Published:** 2018-09-26

**Authors:** Lana Hamieh, Toni K. Choueiri, Barbara Ogórek, Damir Khabibullin, Daniel Rosebrock, Dimitri Livitz, Andre Fay, Jean-Christophe Pignon, David F. McDermott, Neeraj Agarwal, Wenhua Gao, Sabina Signoretti, David J. Kwiatkowski

**Affiliations:** 1 Department of Medicine, Brigham and Women’s Hospital, Boston, MA, United States of America; 2 Department of Medicine, Harvard Medical School, Boston, MA, United States of America; 3 Department of Medical Oncology, Dana-Farber Cancer Institute, Boston, MA, United States of America; 4 Cancer Program, Broad Institute of Harvard and MIT, Cambridge, MA, United States of America; 5 Department of Medical Oncology, PUCRS School of Medicine, Porto Alegre, Brazil; 6 Department of Pathology, Brigham and Women’s Hospital, Boston, MA, United States of America; 7 Department of Medicine, Beth Israel Deaconess Medical Center, Boston, MA, United States of America; 8 Division of Medical Oncology, University of Utah Huntsman Cancer Institute, Salt Lake City, UT, United States of America; Cornell University, UNITED STATES

## Abstract

The mechanistic target of rapamycin (mTOR) is an established therapeutic target in renal cell carcinoma (RCC). Mechanisms of secondary resistance to rapalog therapy in RCC have not been studied previously. We identified six patients with metastatic RCC who initially responded to mTOR inhibitor therapy and then progressed, and had pre-treatment and post-treatment tumor samples available for analysis. We performed deep whole exome sequencing on the paired tumor samples and a blood sample. Sequence data was analyzed using Mutect, CapSeg, Absolute, and Phylogic to identify mutations, copy number changes, and their changes over time. We also performed in vitro functional assays on PBRM1 in RCC cell lines. Five patients had clear cell and one had chromophobe RCC. 434 somatic mutations in 416 genes were identified in the 12 tumor samples. 201 (46%) of mutations were clonal in both samples while 129 (30%) were acquired in the post-treatment samples. Tumor heterogeneity or sampling issues are likely to account for some mutations that were acquired in the post-treatment samples. Three samples had mutations in *TSC1*; one in *PTEN*; and none in *MTOR*. *PBRM1* was the only gene in which mutations were acquired in more than one post-treatment sample. We examined the effect of *PBRM1* loss in multiple RCC cell lines, and could not identify any effect on rapalog sensitivity in *in vitro* culture assays. We conclude that mTOR pathway gene mutations did not contribute to rapalog resistance development in these six patients with advanced RCC. Furthermore, mechanisms of resistance to rapalogs in RCC remain unclear and our results suggest that PBRM1 loss may contribute to sensitivity through complex transcriptional effects.

## Author summary

Mammalian target of rapamycin (mTOR) inhibitors, everolimus and temsirolimus, are FDA-approved for treatment of metastatic renal cell carcinoma (mRCC), but molecular mechanisms of acquired or secondary resistance to these agents are unknown. We evaluated six mRCC patients with available pre-treatment specimens who were treated with mTOR inhibitors and had a good clinical outcome, and then had a second biopsy at the time of resistance. We found that mutations in *PBRM1* appeared to be enriched in post-treatment samples. However, modulation of *PBRM1* levels in vitro in cell lines had no apparent effect on rapalog sensitivity. We conclude that mechanisms of resistance to rapalog therapy in RCC are not easily explained by gene mutations in most cases, and may depend on more subtle transcriptional and/or epigenetic changes.

## Introduction

Both everolimus and temsirolimus, analogs of rapamycin termed rapalogs, are FDA-approved and in common used for treatment of metastatic RCC based on seminal randomized clinical trials [1–3]. However, these drugs are known to cause disease stabilization in most cases, with a 5% objective response rate by standard RECIST criteria.

The Phosphatidylinositol 3-kinase (PI3K)/AKT/mechanistic target of rapamycin (mTOR] pathway plays a critical role in cell growth, differentiation, survival and metabolism. It is frequently activated in a variety of cancer types [4], and new uses of rapalogs in combination with other therapies continue to be discovered [5,6].

mTOR is a serine threonine kinase which occurs in cells in two large multi-component complexes termed mTORC1 and mTORC2 [7,8]. mTORC1 is negatively regulated by the TSC protein complex which consists of TSC1, TSC2, and TBC1D7, which converts the small GTPase RHEB into its inactive GDP-bound form. When both alleles of either *TSC1* or *TSC2* are mutated or lost, as is the rule in tumors occurring in individuals with the genetic disorder tuberous sclerosis complex, RHEB-GTP levels are high, leading to activation of mTORC1. mTORC1 activity is also regulated by PI3K, AKT, MAPK, AMPK, growth factors, nutrient availability, stress levels and oxygen levels. Activation of mTORC1 leads to protein synthesis, lipid synthesis, nucleotide synthesis, autophagy inhibition, leading to cell enlargement and preparation for cell division [9]. Somatic mutations in *MTOR* which deregulate and activate its kinase [10,11] are known to occur in several cancer types, predominantly RCC in which mutation is seen in about 5% [12]. Activating RHEB mutations which activate mTORC1 are quite rare but also known to occur in cancer [13].

Rapalogs are allosteric inhibitors of mTORC1 through binding to FKBP12, which binds to a specific domain of mTORC1 to inhibit its kinase activity. Previously we have reported that response to rapalog therapy in RCC is associated with mutation in the mTOR pathway genes: *TSC1*, *TSC2*, and *MTOR* [14]. Another recent study reported that mutations in *PBRM1* were associated with response to rapalog therapy in the RECORD-3 trial [15]. To our knowledge no previous study has examined molecular mechanisms of acquired or secondary resistance of rapalog therapy in responding patients with RCC.

## Results

### Patient characteristics

We identified six mRCC patients who developed resistance to rapalog therapy after initial clinical benefit, and who had available pre-treatment and post-treatment tumor samples. Five of these 6 patients had been studied in our earlier analysis of the association between mTOR pathway mutations and response to rapalogs in RCC [14]. (However, note that the earlier study did not include analysis of post-treatment as well as pre-treatment samples, and was only gene panel sequencing, not whole exome sequencing.) Five patients had clear cell RCC (ccRCC) and one had chromophobe RCC (Table 1). Most patients had received prior treatment with vascular endothelial growth factor targeted therapies (n = 5) and received a rapalog in the second (n = 3) or third (n = 2) line setting. Five patients received treatment with everolimus and one with temsirolimus. The patients received rapalogs for a median of 9.5 months (range: 5.5–46 months), after which they progressed. One (the chromophobe RCC) had a complete response, four had a partial response, and one had 10% tumor shrinkage (Stable Disease).

**Table 1:** Patient characteristics.

Sample	Age (years)	Gender	Histology	Drug	Treatment line	Treatment duration (months)	Best response
MT-002	68	F	Clear Cell	Temsirolimus	Second	8	PR
MT-003	73	M	Clear Cell	Everolimus	Second	8	SD*
MT-004	61	M	Clear Cell	Everolimus	Second	46	PR
MT-005	50	M	Clear Cell	Everolimus	Third	5.5	PR
MT-006	51	F	Clear Cell	Everolimus	Third	11	PR
MT_007	43	M	Chromophobe	Everolimus	First	27.6	CR

*SD: 10% shrinkage

### Mutational analysis

434 somatic variants were identified in these six patients’ biopsies, including both pre-treatment and post-treatment samples (S1 Table). The mutation profiles of the six individual tumors matched well with the expected genes and mutations from past studies in RCC. Three patients’ tumors showed mutations in TSC1, while one had a mutation in PTEN, and some of these patients had been included in our previous study showing that there is enrichment for mutations in mTOR pathway genes in RCC patients who respond to rapalog therapy [14]. Recurrent mutations (seen in more than one patients’ samples) were seen in 10 genes (Table 2). *VHL* mutations were seen in all 5 ccRCCs, as expected. *PBRM1* mutations were also seen in all 5 ccRCC samples. Three samples had inactivating mutations in *TSC1*, as noted; three including the chromophobe RCC had *TP53* mutations; while two had *BAP1* mutations. Several genes with recurrent mutations were likely chance events, enhanced by their large size: *DNAH11* (4516aa), *TTN* (34350aa), *PIEZO1* (2521aa), *TRPM6* (2022aa); none are thought to be involved in the pathogenesis of any form of cancer. Furthermore, several of these mutations were silent, also suggesting that they were random events. Recurrent mutations in *PTPRN2* may have also been due to random chance, and one of those was also silent.

**Table 2:** Genes with recurrent mutations seen in 6 RCC samples.

subject	locus	Chr	nt	Variant_Classification	Protein_Change	ref	var	N alt count	N ref count	T alt count	T ref count	TA alt count	TA ref count	T AF	TA AF	var_cluster_classes	CCF1	CCF2	delta
MT_002	BAP1	3	52436384	Frame_Shift_Del	p.V704fs	C	-	0	384	41	408	67	440	0.09	0.13	C_C	1.00	0.95	-0.05
MT_006	BAP1	3	52436388	Frame_Shift_Del	p.Q702fs	T	-	0	386	84	279	0	365	0.23	0.00	S_Z	0.88	0.01	-0.87
MT_005	DNAH11	7	21657291	Missense_Mutation	p.E1389K	G	A	0	105	5	100	0	245	0.05	0.00	C_Z	1.00	0.00	-1.00
MT_006	DNAH11	7	21826340	Silent	p.V3239V	G	T	0	156	10	85	0	267	0.11	0.00	S_Z	0.88	0.01	-0.87
MT_002	PBRM1	3	52620471	Frame_Shift_Del	p.S1119fs	A	-	0	61	7	49	26	147	0.13	0.15	C_C	1.00	0.95	-0.05
MT_003	PBRM1	3	52588861	Silent	p.P1496P	A	G	0	48	0	33	6	34	0.00	0.15	Z_C	0.02	1.00	0.98
MT_004	PBRM1	3	52696199	Nonsense_Mutation	p.E160*	C	A	0	42	13	42	15	49	0.24	0.23	C_C	1.00	1.00	0.00
MT_005	PBRM1	3	52702606	Nonsense_Mutation	p.Q98*	G	A	0	40	0	28	23	31	0.00	0.43	Z_C	0.00	1.00	1.00
MT_006	PBRM1	3	52643360	Nonsense_Mutation	p.E846*	C	A	0	24	0	13	6	50	0.00	0.11	Z_C	0.02	1.00	0.98
MT_002	PIEZO1	16	88783268	Silent	p.I2233I	C	T	0	318	0	380	76	400	0.00	0.16	Z_C	0.07	0.97	0.90
MT_003	PIEZO1	16	88799715	Missense_Mutation	p.P879T	G	T	0	98	0	163	21	149	0.00	0.12	Z_C	0.02	1.00	0.98
MT_006	PTPRN2	7	157341689	Missense_Mutation	p.A976V	G	A	0	259	0	193	18	352	0.00	0.05	Z_S	0.02	0.22	0.20
MT_007	PTPRN2	7	157997958	Silent	p.T95T	G	A	0	83	10	156	1	170	0.06	0.01	S_Z	0.15	0.03	-0.12
MT_005	TP53	17	7578534	Missense_Mutation	p.K132N	C	A	0	157	11	138	0	245	0.07	0.00	C_Z	1.00	0.00	-1.00
MT_006	TP53	17	7578191	Missense_Mutation	p.Y220H	A	G	1	283	0	210	46	306	0.00	0.13	Z_C	0.02	1.00	0.98
MT_007	TP53	17	7574018	Missense_Mutation	p.R337C	G	A	0	162	97	32	56	127	0.75	0.31	C_C	0.96	0.96	0.00
MT_005	TRPM6	9	77442779	Silent	p.D252D	A	G	0	107	8	135	0	352	0.06	0.00	C_Z	1.00	0.00	-1.00
MT_006	TRPM6	9	77411766	Missense_Mutation	p.E761A	T	G	2	177	16	47	0	206	0.25	0.00	S_Z	0.88	0.01	-0.87
MT_002	TSC1	9	135778013	Nonsense_Mutation	p.Y790*	G	C	0	159	8	143	1	391	0.05	0.00	S_Z	0.60	0.01	-0.59
MT_004	TSC1	9	135797317	Frame_Shift_Del	p.V184fs	C	-	0	122	39	160	54	172	0.20	0.24	C_C	1.00	1.00	0.00
MT_006	TSC1	9	135797269	Frame_Shift_Del	p.V200fs	G	-	0	223	0	125	50	417	0.00	0.11	Z_C	0.02	1.00	0.98
MT_004	TTN	2	179514942	In_Frame_Del	p.IAPEEE11604del	TTTCCTCTTCAGGAGCAA	-	0	12	7	50	3	44	0.12	0.06	C_C	1.00	1.00	0.00
MT_006	TTN	2	179511275	Missense_Mutation	p.E11762K	C	T	0	92	0	22	7	86	0.00	0.08	Z_C	0.02	1.00	0.98
MT_002	VHL	3	10188261	Nonsense_Mutation	p.L135*	T	A	0	265	27	172	81	318	0.14	0.20	C_C	1.00	0.95	-0.05
MT_003	VHL	3	10188240	Missense_Mutation	p.L128P	T	C	0	338	100	189	41	261	0.35	0.14	C_C	1.00	1.00	0.00
MT_004	VHL	3	10183752	In_Frame_Del	p.74_	TCATCTTCT	-	0	241	37	323	17	203	0.10	0.08	C_C	1.00	1.00	0.00
MT_005	VHL	3	10188277	Frame_Shift_Ins	p.N141fs	-	A	0	150	12	216	109	282	0.05	0.28	S_S	0.97	0.69	-0.28
MT_006	VHL	3	10191614	Frame_Shift_Del	p.QER203fs	CAGGAGCG	-	0	339	14	123	32	447	0.10	0.07	S_S	0.43	0.59	0.16

N alt count, number of reads with the alternate allele in the normal sample

N ref count, number of reads with the reference allele in the normal sample

T alt count, number of reads with the alternate allele in the pre-treatment tumor sample

T ref count, number of reads with the reference allele in the pre-treatment tumor sample

TA alt count, number of reads with the alternate allele in the post-treatment tumor sample

TA ref count, number of reads with the reference allele in the post-treatment tumor sample

T AF, allele frequency of the alternate allele in the pre-treatment tumor sample

TA AF, allele frequency of the alternate allele in the post-treatment tumor sample

var_cluster_classes, Variant cluster classes: Z, zero; S, subclonal; C, clonal

CCF1, Cancer cell fraction in the pre-treatment tumor sample

CCF2, Cancer cell fraction in the post-treatment tumor sample

Delta, = CCF2 - CCF1

### Clonality analysis

ABSOLUTE was used to calculate the tumor purity of the samples, and relative allelic frequency of each mutation in the cancer cells, termed clonal cell fraction (CCF) [16–18]. Phylogic was used to construct phylogenetic trees for the pre- and post-treatment pairs of samples, and to generate diagrams showing the major mutational events and clonal evolution (S1 Fig).

46% of the somatic variants identified were clonal in both the pre-treatment and post-treatment samples, while 25% of variants were not seen in the pre-treatment sample but were clonal in the post-treatment sample (S2 Table). *VHL* mutations were clonal or high subclonal in all 5 ccRCC samples, as expected, and showed no significant change in clonal representation in the paired samples. A *TSC1* mutation went from subclonal to zero in one sample (MT_002) after treatment, consistent with a model in which *TSC1*-mutation bearing cells might have been sensitive to rapalog therapy, and were selectively killed or inhibited by such therapy. However, in another sample (MT_006), an inactivating *TSC1* mutation went from zero cancer cell fraction in the pre-treatment sample to clonal in the post-treatment sample, in direct contrast to this model. Mutations in each of *TP53* and *PTPRN2* also were enriched in one patient’s post-treatment sample but lost in another patient’s post-treatment sample.

Multiple copy number (CN) events were also identified in these tumor samples (S3 Table). All ccRCC samples showed loss of one copy of 3p, as expected, and the chromophobe sample showed loss of multiple chromosomes (1, 2, 6, 8, 10, 11p, 13, 17, 21). There were no focal amplifications identified in the post-treatment samples, but rather a variety of chromosome and arm level gains and losses of uncertain significance.

*PBRM1* mutations were clonal in both pre-treatment and post-treatment samples from two patients, but went from zero to clonal in 3 patients samples’ following treatment. Two of these acquired mutations were nonsense mutations in *PBRM1* while the third was a synonymous change. *PBRM1* mutations are seen in about 30% of ccRCC samples [19], and it is possible that the appearance of PBRM1 mutations in the post-treatment samples was due solely to tumor heterogeneity and/or sampling issues. However, as noted above, VHL mutations were present at or near clonal frequency in all 5 ccRCC samples both pre- and post-treatment. Hence, the finding that *PBRM1* mutations were present in all 5 ccRCC samples at the time of resistance, including acquisition of mutations in 3 ccRCC samples led us to explore the hypothesis that *PBRM1* loss is a mechanism of resistance to rapalog therapy in ccRCC.

### Analysis of *PBRM1* as a candidate mediator of resistance to rapalog therapy in ccRCC

**Fig 1:**
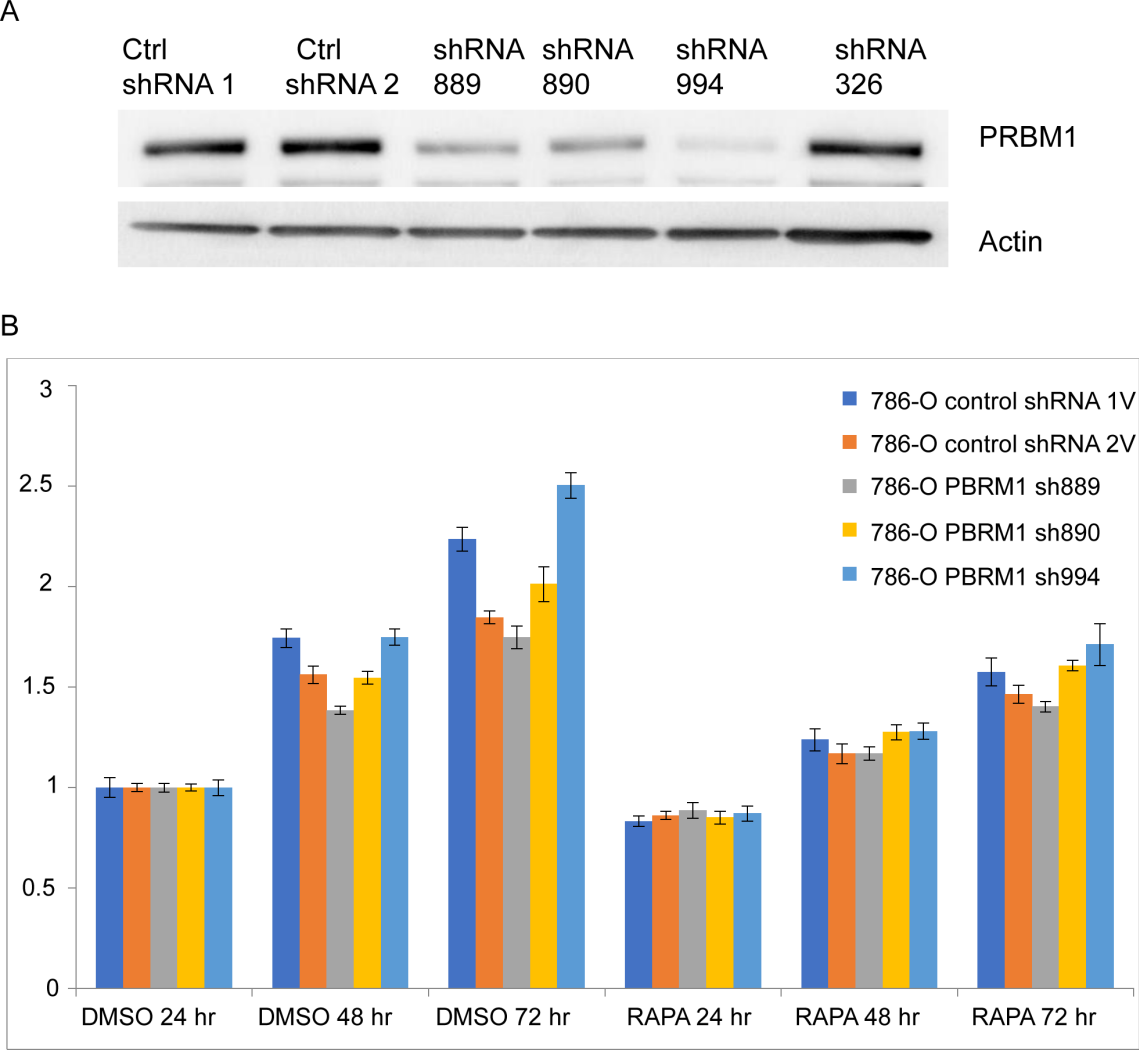
PBRM1 downregulation has no effect on growth inhibition by rapamycin in the ccRCC line 786-O. (**A**) PBRM1 expression in 786-O cells stably expressing different *PBRM1* shRNAs was assessed by immunoblotting. **(B**) 786-O cells stably expressing control shRNAs or *PBRM1* shRNAs 889, 890 and 994 were treated with DMSO or 20nM rapamycin (RAPA) for 24, 48 and 72hr. Cell number was quantified using Crystal Violet, and normalized to day 1 (24 hr).

We studied several ccRCC cell lines with native wild type (786-O, SNU-349) or native mutant PBRM1 (A704, RCC4) [20–22]. In 786-O cells, 4 different shRNA clones (sh889, sh890, sh994, sh326) were used to generate stable lines with reduced *PBRM1* expression (Fig 1A). The 3 lines with greatest knock-down showed somewhat variable growth rates in comparison to a similarly derived line expressing a control shRNA, and all lines showed moderate growth inhibition in response to rapamycin treatment at 20 nM for up to 3 days with no difference between the PBRM1 knock-down cells and those expressing control shRNAs (Fig 1B). Similar results were obtained from SNU349 cells with stable downregulation of PBRM1 (S2 Fig).

We also studied RCC4 cells that are known to have biallelic mutation in PBRM1 that leads to a complete loss of expression of the protein [21]. We used derivative RCC4 cell lines expressing either control vector or wild type VHL [20]. We observed significant growth inhibition of both RCC4-vector and RCC4-VHL cells in response to each of rapamycin and Torin1 in 96well plate assays (S3 Fig). This also suggested that PBRM1 loss did not lead to rapamycin resistance.

**Fig 2:**
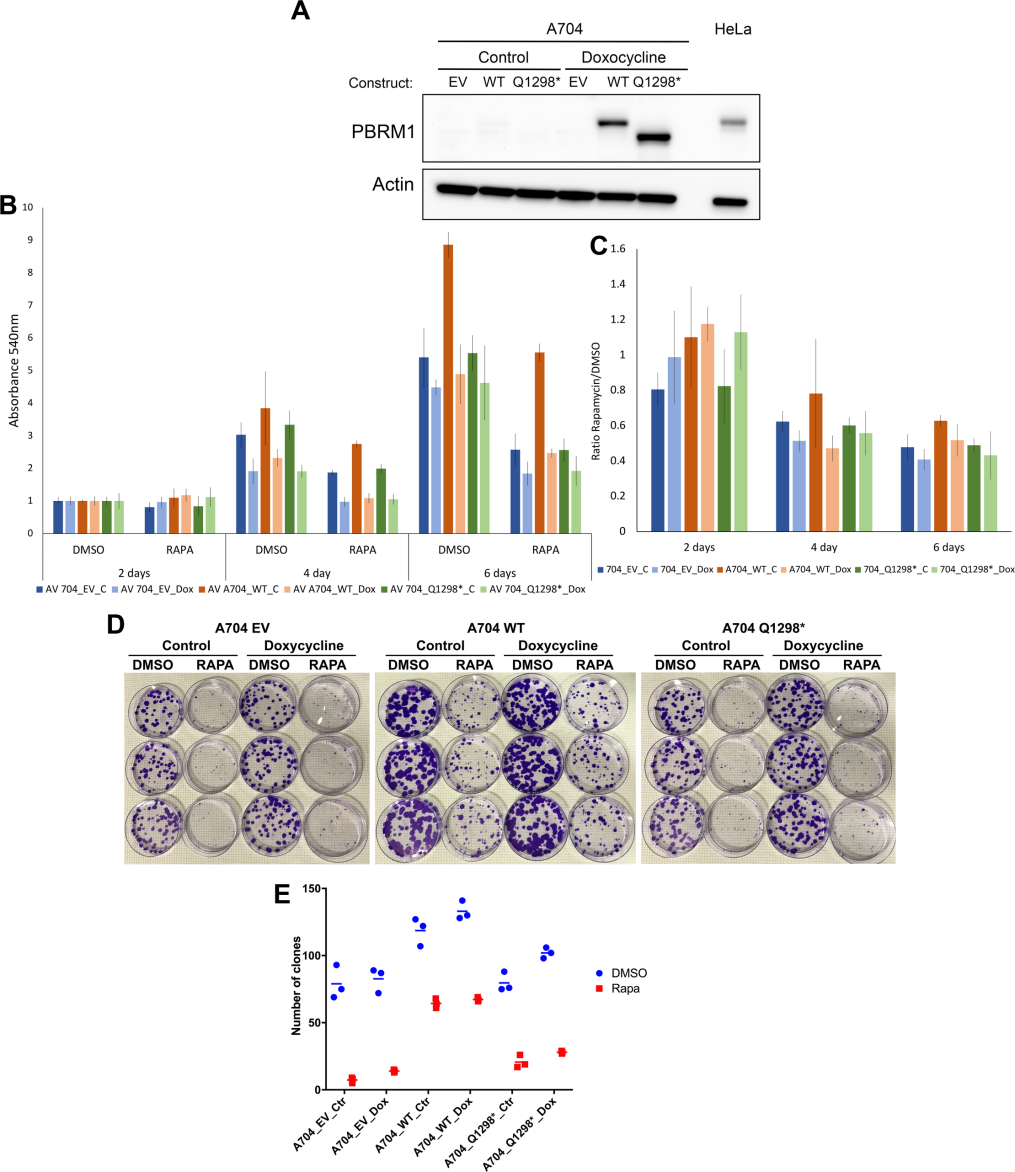
Rapamycin has similar effects in growth inhibition of RCC 704 cells independent of PBRM1 addback. (**A**) Immunoblot analysis of the PBRM1 null A704 cell line stably transfected with doxycycline inducible constructs expressing empty vector (EV), wild-type PBRM1 (WT) or mutant Q1298* PBRM1, treated with or without doxycycline (Dox, 1ug/ml) for 5 days. (**B**) Growth of these same A704 derivative cell lines treated with or without doxycycline and with DMSO (control) or rapamycin (RAPA, 20nM) for 2, 4, or 6 days. Cell growth was assessed by Crystal Violet staining. (**C**) Proportional growth of A704 derivative cell lines derived from panel B. Rapamycin inhibited the growth of all 3 cell lines to a similar extent, and doxycycline treatment had little or no effect on growth. (**D, E**) These same A704 derivative cell lines were plated at 200 cells per 10cm dish and treated with or without Doxycycline and DMSO control or Rapamycin (20nM) for 30 days, then formalin fixed and stained with Crystal Violet. **D**, Image of colonies after crystal violet staining. **E**, Quantitation of colonies > 2mm in diameter per 10 cm dish. The bar is placed at the median value.

We also performed the converse experiment, in that we examined rapalog sensitivity of derivatives of a native PBRM1 null cell line (A704) expressing either empty vector (A704_EV), wild type PBRM1 (A704_WT), or a mutant Q1298* PBRM1 (A704_Q1298*) under regulation of doxycycline [23] (Fig 2A). There were minor differences in the growth rate of these various A704 derivative lines, but there was no appreciable difference among them in response to rapamycin treatment for up to 6 days with and without doxycycline induction (Fig 2B and 2C). We also examined the growth of these various A704 sublines in a clonogenic assay under rapamycin treatment for 30 days (Fig 2D and 2E). Different numbers of colonies were seen for the different A704 derivative lines that varied to a small extent with and without doxycycline. All three lines showed a significant reduction in colony growth in response to rapamycin, and for the A704_WT line, a similar reduction in colony number was seen with and without doxycycline induction of wild type PBRM1.

### Examination of other genes in which mutations correlated with resistance

One hundred twenty-nine genes showed a significant increase in the cancer cell fraction for a single mutation in a single post-treatment tumor sample in comparison to the pre-treatment sample (S4 Table). To assess whether these genes were enriched in a pathway that might be consistent with resistance to rapalogs, we performed gene set enrichment analysis using hallmark gene sets [24]. The 129 genes showed modest overlap with two hallmark gene sets (E2F targets, and mitotic spindle), 4 genes each with FDR q = 0.044, and no enrichment for any other hallmark gene set. None of these genes were obvious members of the PI3K-AKT-mTOR signaling cascade. Some were known cancer genes, including *CDKN2A*, *KEAP1*, *MYCN*, *PLK4*, *SETD2*, *TP53*. It is possible that any of these singleton genetic changes contributed to resistance to rapalog therapy in an individual patient.

## Discussion

In this study, we sought to identify molecular mechanisms of secondary resistance to rapalog therapy in patients with RCC who had demonstrated initial clinical benefit (5 of 6 had PR/CR). Our analyses were limited by both the relatively small number of samples available to us (n = 6), and that only one of the six patients had experienced a durable CR, whereas four had PRs only, and one had prolonged stable disease.

Furthermore, tumor heterogeneity is well-known in RCC [12,25–27], and complicates interpretation of genetic differences seen in the two paired tumor samples. 63 to 69% of all somatic mutations were not detectable across every tumor region of RCC when multiple samples were analyzed [25], similar to our findings that 46% of somatic mutations were not seen in both of our paired samples for these 6 patients (S2 Table). Consequently the 129 genes with somatic mutations that were enriched (CCF increased by > 0.5) in a single post-treatment sample likely reflect that heterogeneity, and are each unlikely to contribute to rapalog resistance. However, it is possible that some of those ‘acquired’ mutations seen only in the post-treatment samples may have contributed to resistance.

*PBRM1* was the only gene to show gain of mutation in more than one patient in the post-treatment sample. Two ccRCC patients showed gain of a nonsense mutation in *PBRM1*, while a third showed gain of a synonymous mutation, and all three were clonal in the post-treatment sample. However, through analysis of RCC cell lines with both native *PBRM1* expression, and those with native loss of *PBRM1*, we could find no evidence of differential sensitivity to rapamycin therapy in standard and clonal growth assays at the standard dose of rapamycin 20nM, which is similar to serum trough levels of this compound achieved in patients with standard dosing, 10-15nM. It remains possible that the effects of *PBRM1* loss with respect to rapalog sensitivity are not modeled well in tissue culture systems, and that this genetic event still contributed to rapalog resistance in patients. Alternatively, tumor heterogeneity or sampling issues may account for these PBRM1 mutations seen only in the post-treatment samples.

Interestingly, Hsieh et al. reported that *PBRM1* mutations were associated with longer progression free survival (PFS) in metastatic RCC patients treated with first-line everolimus in the RECORD-3 trial [15]. Hence it is possible that the finding of *PBRM1* mutations in our 5 ccRCC patients correlates with response to rapalog therapy, though not seen in the pre-treatment tumor specimen, rather than representing a mechanism of resistance. On the other hand, if PBRM1 mutations cause response, then loss of PBRM1 mutation might be expected in the post-treatment resistance sample, given tumor heterogeneity in RCC, and we did not observe this.

We were somewhat surprised that we did not identify any secondary mutations in *MTOR* in these samples. Previous studies have identified *MTOR* mutations capable of preventing each of rapalog and ATP-competitive kinase inhibition of mTOR kinase activity in vivo and in vitro [28,29]. Furthermore, a variety of activating mutations in *MTOR* are well-known in both RCC and other cancer types [10–12,27,30], and in some cases are associated with exceptional response to rapalog therapy. Nonetheless, no *MTOR* mutations were seen in any of these six patients, nor were *MTOR* mutations associated with resistance development in even a single case.

Hence, we conclude that mechanisms of resistance to rapalog therapy in RCC are not easily explained by mutations in most cases, and likely depend on more subtle transcriptional and/or epigenetic changes. Transcriptional effects of *PBRM1* mutation have recently been identified in analysis of the association of response of ccRCC to immune checkpoint therapies [31], and may have a similar effect in enhancing response to rapalogs.

## Materials and methods

### Ethics statement

This research study was approved by the Dana Farber/Harvard Cancer Center Office for Human Research Studies, protocol 07–336, and all subjects provided written informed consent.

### Patient selection

We identified patients with metastatic RCC who initially responded to treatment with rapalog for at least 5 months, and then showed progressive disease, with available pre- and post-treatment (at time of progression) biopsies through a search of our own medical facilities and national and international collaborators. The six patients were treated with temsirolimus or everolimus at one of three medical centers: Dana-Farber Cancer Institute, Beth Israel Deaconess Medical Center, and the University of Utah Hospital.

### Sample sequencing and variant analysis

From each patient, we collected 1) a pre-treatment nephrectomy specimen, 2) post-treatment metastatic tumor specimen, and 3) a venous blood specimen. An expert genitourinary pathologist (SS) reviewed hematoxylin and eosin stained slides. For each case regions containing at least an estimated 50% tumor cells were selected for DNA extraction. Selected tumor areas were scraped off unstained slides and DNA was extracted using the QIAamp DNA FFPE Tissue Kit (QIAGEN, Valencia, CA), according to the manufacturer guidelines.

Whole exome sequencing (WES) was performed at the Broad Institute following standard protocols. Sequencing data was analyzed using standard analytic pipelines deployed in the Firehose environment. Mutect and Indelocator were used to identify somatic mutations in tumor-normal pairs. Every single mutation that was called by these pipelines was scrutinized using IGV to assess the reliability of the variant call, and to confirm allele frequencies seen in the various samples. Many variants were discarded due to misaligned reads, repetitive sequence tracts, low quality base or read scores, or reads seen in only a single direction.

Recapseg and AllelicCapseg were used to determine copy number profiles. ABSOLUTE was used to estimate sample purity and ploidy, absolute copy number for each chromosome and segment, and clonal cell fraction (CCF) values for each mutation [16,17]. Phylogic was used to perform Bayesian clustering of mutation CCFs, and to construct phylogenetic trees for the pre- and post-treatment samples, as described previously [17,18].

### Cell line studies

We studied RCC cell lines SNU349, 786-O, RCC4, and several versions of the A704 cell line (A704+BAF180_WT, A704+BAF180_Q1298*, A704+BAF180_EV) previously generated by one of the co-authors (WG) [19]. Stable PBRM1 knock down was performed using four different shRNAs (Sigma) in lentiviruses following standard methods; reduced PBRM1 expression was confirmed by SDS-PAGE and immunoblotting of cell lysates. Cell growth assays were performed for the indicated time points in clear 96-well plates for Crystal Violet staining or white opaque 96-well plates (Corning) for cellular ATP measurement using Cell Titer Glo (Promega). Rapamycin was used at 20nM and compared with vehicle (DMSO) treatment. Clonogenic cell proliferation assays were performed by plating 200 cells in 10 cm dishes (n = 3 for each cell line/condition), treating them every 3 days with Rapamycin (20nM) or corresponding DMSO control, with and without doxycycline (1ug/ml) for 30 days, and then counting visible colonies without magnification following Crystal Violet staining.

## Supporting information

S1 Table All mutations seen in 6 RCC samples either pre-treatment or post-treatment with rapalog. (XLSX)

S2 Table Clinical features, sequencing metrics, and clonality transitions in pre- and post-treatment samples from 6 RCC patients. (XLSX)

S3 Table Copy number events seen in pre-treatment and post-treatment samples. (XLSX)

S4 Table Genes and variants showing a significant increase in cancer cell fraction (> 0.5) in a post-treatment sample. (XLSX)

S1 Fig Phylogenetic tree diagrams for 6 RCCs produced using Phylogic. A-F. Diagrams for each of the six RCC samples studied here. Truncal clonal mutations present in both pre- and post-treatment samples are shown along the wide gray trunk extending from normal tissue (clear circle) to initial tumor clone (grey circle). Mutations seen only in the pre-treatment sample are shown on the line extending to the blue circles. Mutations seen only in the post-treatment sample are shown on the line extending to the red and brown circles. Specifically, the color of the lines and circles reflect mutations that change in allele frequency from pre-treatment to post-treatment as follows: red, zero to clonal; brown, zero to subclonal; purple, subclonal to clonal; dark blue, subclonal to zero; light blue, clonal to zero; gray clonal to clonal. The only mutations that are shown are those in known cancer genes.
(DOCX)

S2 Fig PBRM1 downregulation has no effect on growth inhibition by rapamycin in the ccRCC line SNU-349. (A) PBRM1 expression in SNU-349 cells stably expressing different PBRM1 shRNAs was assessed by immunoblotting. (B) SNU-349 cells stably expressing control shRNAs or PBRM1 shRNAs 890 and 994 were treated with DMSO or 20nM rapamycin (RAPA) for 24, 48 and 72hr. Cell proliferation was quantified using Cell Titer Glo, and normalized to day 1 (24 hr).
(DOCX)

S3 Fig Effects of rapamycin treatment on RCC4 cells with and without VHL addback. RCC4-vector and RCC4-VHL cells were treated with DMSO or 20nM rapamycin (RAPA) or 250nM Torin1 for 24, 48 and 72hr. Cell number was quantified using Crystal Violet, and normalized to day 1 in DMSO (24 hr).
(DOCX)
