# Supplementary material for: Mechanisms of acquired resistance to rapalogs in metastatic renal cell carcinoma
Source: PLoS Genet. 2018 Sep 26;14(9):e1007679. doi: 10.1371/journal.pgen.1007679 (PMC6181431; doi:10.1371/journal.pgen.1007679)
Supplement: S1 Fig — A-F. Diagrams for each of the six RCC samples studied here. Truncal clonal mutations present in both pre- and post-treatment samples are shown along the wide gray trunk extending from normal tissue (clear circle) to initial tumor clone (grey circle). Mutations seen only in the pre-treatment sample are shown on the line extending to the blue circles. Mutations seen only in the post-treatment sample are shown on the line extending to the red and brown circles. Specifically, the color of the lines and circles reflect mutations that change in allele frequency from pre-treatment to post-treatment as follows: red, zero to clonal; brown, zero to subclonal; purple, subclonal to clonal; dark blue, subclonal to zero; light blue, clonal to zero; gray clonal to clonal. The only mutations that are shown are those in known cancer genes. (DOCX) [file pgen.1007679.s005.docx]

Figure S1. Phylogenetic tree diagrams for 6 RCCs produced using Phylogic. Truncal clonal mutations present in both pre- and post-treatment samples are shown along the wide gray trunk extending from normal tissue (clear circle) to initial tumor clone (grey circle). Mutations seen only in the pre-treatment sample are shown on the line extending to the blue circles. Mutations seen only in the post-treatment sample are shown on the line extending to the red and brown circles. Specifically, the color of the lines and circles reflect mutations that change in allele frequency from pre-treatment to post-treatment as follows: red, zero to clonal; brown, zero to subclonal; purple, subclonal to clonal; dark blue, subclonal to zero; light blue, clonal to zero; gray clonal to clonal. The only mutations that are shown are those in known cancer genes.

A) MT_002 B) MT_003

C) MT_004 D) MT_005

E) MT_006 F) MT_007
