# Supplementary material for: Mechanisms of acquired resistance to rapalogs in metastatic renal cell carcinoma
Source: PLoS Genet. 2018 Sep 26;14(9):e1007679. doi: 10.1371/journal.pgen.1007679 (PMC6181431; doi:10.1371/journal.pgen.1007679)
Supplement: S2 Fig — (A) PBRM1 expression in SNU-349 cells stably expressing different PBRM1 shRNAs was assessed by immunoblotting. (B) SNU-349 cells stably expressing control shRNAs or PBRM1 shRNAs 890 and 994 were treated with DMSO or 20nM rapamycin (RAPA) for 24, 48 and 72hr. Cell proliferation was quantified using Cell Titer Glo, and normalized to day 1 (24 hr). (DOCX) [file pgen.1007679.s006.docx]

Figure S2. PBRM1 downregulation has no effect on growth inhibition by rapamycin in the ccRCC line SNU-349. (**A**) PBRM1 expression in SNU-349 cells stably expressing different *PBRM1* shRNAs was assessed by immunoblotting. **(B**) SNU-349 cells stably expressing control shRNAs or *PBRM1* shRNAs 890 and 994 were treated with DMSO or 20nM rapamycin (RAPA) for 24, 48 and 72hr. Cell number was quantified using Crystal Violet, and normalized to day 1 (24 hr).


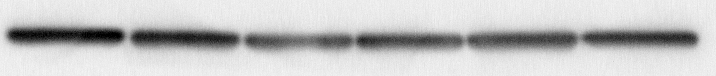

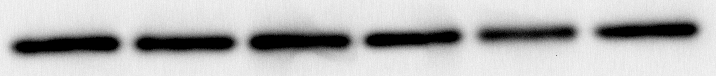

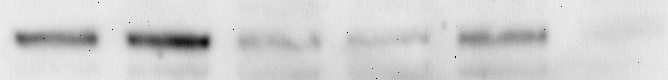


PBRM1

pS6 (S235/236)

actin

Ctrl 1V

Ctrl 2V

sh889

sh890

sh993

sh994

SNU-349 cells
