# Supplementary material for: Mechanisms of acquired resistance to rapalogs in metastatic renal cell carcinoma
Source: PLoS Genet. 2018 Sep 26;14(9):e1007679. doi: 10.1371/journal.pgen.1007679 (PMC6181431; doi:10.1371/journal.pgen.1007679)
Supplement: S3 Fig — RCC4-vector and RCC4-VHL cells were treated with DMSO or 20nM rapamycin (RAPA) or 250nM Torin1 for 24, 48 and 72hr. Cell number was quantified using Crystal Violet, and normalized to day 1 in DMSO (24 hr). (DOCX) [file pgen.1007679.s007.docx]

Figure S3. Effects of rapamycin treatment on RCC4 cells with and without VHL addback. RCC4-vector and RCC4-VHL cells were treated with DMSO or 20nM rapamycin (RAPA) or 250nM Torin1 for 24, 48 and 72hr. Cell number was quantified using Crystal Violet, and normalized to day 1 in DMSO (24 hr).
